# A Comparison of Social Exclusion Towards People with Depression or Chronic Back Pain

**DOI:** 10.1177/20494637221148337

**Published:** 2023-01-07

**Authors:** Lucy Roberts-West, Amy Gravatt, Natasha Guest, Ashley Hunt, Laraib Siddique, Danijela Serbic

**Affiliations:** Department of Psychology, 3162 Royal Holloway University of London, Egham, SRY, UK

**Keywords:** public stigma, social exclusion, depression, chronic back pain, empathy

## Abstract

**Objectives:**

Research comparing mental and physical health stigma is scarce. The aim of this study was to compare social exclusion towards hypothetical males and females with depression or chronic back pain. Furthermore, the study investigated whether social exclusion is associated with participant’s empathy and personality traits, while controlling for their sex, age and personal exposure to mental/physical chronic health conditions.

**Design:**

This study employed a cross-sectional questionnaire design.

**Methods:**

Participants (*N* = 253) completed an online vignette-based questionnaire and were randomly allocated to either a depression or chronic back pain study condition. Measures of social exclusion through respondents’ willingness to interact with hypothetical individuals, empathy and the Big Five personality traits were completed.

**Results:**

Willingness to interact scores did not significantly differ depending on the diagnosis or sex of the hypothetical person in the vignette. For depression, higher levels of conscientiousness significantly predicted less willingness to interact. Whilst being a female participant and having higher empathy significantly predicted greater willingness to interact. For chronic back pain, higher empathy significantly predicted greater willingness to interact, with no significant predictors found from the Big Five personality traits.

**Conclusion:**

Findings indicate that females and males with depression or chronic back pain face similar levels of social exclusion, with empathy being a core variable driving social exclusion behaviours. These findings enhance our understanding of potential variables driving social exclusion, in-turn informing campaign development to reduce public stigma towards depression and chronic back pain.

Stigma is defined as an attribute that it is deeply discrediting^1^ and is argued to have two major dimensions: public stigma and self-stigma.^2^ Public stigma is a construct comprising of beliefs (stereotypes), emotional reactions (prejudice) and behaviours (discriminatory behaviours), resulting in the general public attributing negative responses towards others.^3^ The present study will focus on public stigma, more specifically an aspect of discriminatory behaviour termed social exclusion. Social exclusion reflects attitudes towards others through minimised willingness to interact, such as reducing social distance and interaction with others.^4^ Stigma is recognised to be a causal ‘driver’ of social exclusion behaviours towards mental illness, impacting individual’s well-being, mortality, employment and participation in healthcare.^5–7^ Through improving knowledge of social exclusion towards different health conditions, one can better aim to minimise the impact of social exclusion through intervention, underlining the importance of this research.

It is well established that public stigma, including social exclusion, exists towards mental health^8^ and physical health conditions.^9^ The present study has a specific focus on two common health problems: depression and chronic back pain. Depression is one of the most prevalent mental health conditions with an estimated 300 million people having depression in 2015.^10^ Chronic back pain is one of the most prevalent physical health conditions experienced by 50–80% of adults at some point in their life^11^ and is the leading cause of disability globally,^12^ significantly reducing quality of life.^13^ Both conditions face high levels of public stigma and social exclusion.^9,14–22^ Individuals with depression are often perceived as dangerous, impaired, and unpredictable,^23,24^ creating barriers to treatment.^16,25^ Whilst the medical invisibility of chronic pain creates a vicious cycle between chronic pain and stigmatisation,^26,27^ leaving individuals feeling that their pain is imagined or not credible.^21,28,29^ This can result in feelings of guilt, associated with greater pain intensity and disability.^21^

Studies are yet to compare social exclusion towards mental health and chronic pain conditions. An exception to this is the Naushad et al.^30^ study which reported that participants with chronic pain perceived less stigma for their condition than participants with depression. Compared to participants with chronic pain alone, people with both chronic pain and depression perceived more stigma of chronic pain suggesting depression may exacerbate perceived stigma felt by individuals with chronic pain conditions. Recent findings also report that people with pain are significantly more likely to report discrimination than individuals without pain, and the experience of discrimination is associated with increased depression.^31^ A greater understanding is needed regarding what factors drive social exclusion towards depression and chronic low back pain, including the characteristics of both the stigmatised and stigmatising individual.

One such factor is whether males and females face different levels of social exclusion. Participants are more willing to interact with hypothetical females than males with depression as males with depression are seen as more responsible for their symptoms and more likely to cause harm.^32^ Yet, Newton et al.^33^ reported that females with chronic pain are typically stigmatised more than males, falling victim to negative stereotypes as they are perceived as more emotionally expressive and prone to seeking medical help for trivial reasons, leading to others disbelieving their pain. Further evidence is needed to support these claims.

Research has also investigated participant’s empathy and stigma. Empathy can be defined as the ability to put oneself into the shoes of another, understanding and feeling their experience.^34^ Empathy has been reported to have a significant negative relationship with social responsibility, an element of stigma, towards people with depression.^35^ Empathy has also been suggested to act as positive attribute reducing stigma towards people with chronic pain in medical care.^36,37^ The relationship between empathy and social exclusion towards chronic pain is unknown in the general population.

A limited number of studies have investigated the relationship between personality and social exclusion, needing further research. The personality traits of agreeableness and openness to experience from the Big Five Factor Model^38^ have been found to negatively associate with mental health stigma.^39–41^ Thus, suggesting these traits are key in forming less stigmatising attitudes. Yuan et al.^41^ also reported that extraversion was only positively associated with the weak-not-sick dimension of stigma, reflecting views that illness is a personal weakness controlled by the individual. Conscientiousness and neuroticism were only positively associated with the social distance dimension of stigma, reflecting an individual’s desire to avoid contact with another individual. Findings suggest that these traits are important regarding specific aspects of mental health stigma. The relationship between the Big Five and chronic pain stigma is unclear, and this area of research is critical in understanding how social exclusion develops and hence can be reduced.

It is unclear how age influences social exclusion towards chronic pain, but in mental health research findings generally report that younger people are less stigmatising of depression and mental illness,^42–45^ albeit with some exceptions.^46,47^ Mental health literature also suggested that females are less likely to stigmatise depression than males.^45,48–52^ It is possible similar findings may be found within chronic pain research using the general population, although no significant relationship has been found between sex and public stigma in chronic pain using nursing students.^53^ Participants who have had personal experience of depression report significantly lower levels of stigma towards mental health illnesses than those who lack personal experience of depression.^45,54^ Therefore, experiencing illness first-hand is likely to influence attitudes towards illness.

A greater understanding of how social exclusion differs towards people with depression and chronic back pain, and the factors associated with social exclusion, could provide invaluable information to develop effective stigma-based prevention strategies. Therefore, the present study has two key aims: (1) To investigate social exclusion through measurement of willingness to interact, towards hypothetical males and females with either depression or chronic back pain; and (2) examine whether social exclusion is associated with participant’s empathy and personality traits, while controlling for their sex, age and exposure to mental/physical chronic health conditions.

We proposed the following hypotheses based on the existing literature: Respondents will be significantly less willing to interact with hypothetical individuals diagnosed with depression than chronic back pain. Hypothetical males in the vignette will experience lower levels of willingness to interact from respondents than hypothetical females. Empathy, agreeableness and openness will be positively associated with willingness to interact scores towards hypothetical individuals with depression and chronic back pain while controlling for participant’s sex, age and personal exposure to mental/physical chronic health conditions. Extraversion, conscientiousness and neuroticism will be negatively associated with willingness to interact scores towards hypothetical individuals with depression and chronic back pain while controlling for participant’s sex, age and personal exposure to mental/physical chronic health conditions.

## Methods

### Participants

Opportunity sampling was used to recruit participants through advertisements on authors’ social media accounts including websites such as Facebook and Instagram to gain nationwide coverage of the population in the United Kingdom. Inclusion criteria included participants over the age of 18 who had full completion of the questionnaire. Exclusion criteria included participants under the age of 18 and/or who did not complete the questionnaire in full. A total of 302 responses were collected with 45 participants removed from analysis due to omission of most questions, a common issue with online data collection, and four for being under the age of 18. The sample therefore consisted of 253 participants (83 males, 170 females; age: M = 28.67, SD = 14.61). The study was granted ethical approval by the university (Royal Holloway), number: Full-Review-693–2019-11–29-11–00-URJT279.

### Measures

Participants completed an online questionnaire, built using Qualtrics,^55^ which took approximately 12 minutes to complete. To determine their personal experience of the diagnosis included in the vignette, participants were asked: ‘Do you have any personal experience with any mental health conditions (for depression study conditions)/physical chronic health conditions (for chronic back pain study conditions)?’.

This was followed by seven items from the dimension ‘Willingness to interact with the person in the scenarios’ from the Third National Scottish survey of public attitudes to mental health, mental wellbeing and mental health problems, to measure respondent’s social exclusion behaviours.^32^ This social distance scale measures respondents’ willingness to interact with a hypothetical individual in several scenarios, reflecting respondents’ social exclusion behaviour. Willingness to interact is a common method used to measure social exclusion behaviours towards clinical conditions, including chronic pain conditions, at the general population level.^56,57^ Specifically, participants indicated their willingness to interact with the described person on a 5-point Likert scale ranging from 1 (*very unwilling*) to 5 (*very willing*). The scale produces a continuous score of participant’s willingness to interact. Scores are summed with high scores indicating greater willingness to interact and less stigma, with a minimum score of 7 and a maximum score of 35. The scale was assumed appropriate to be generalised towards chronic pain conditions as well. An example item includes ‘How willing would you be to move next door to Joe/Ellie?’ with additional items covering topics such as socialising, work and friends. Reliability analysis run on our data demonstrated Cronbach’s alpha was 0.854, meaning this scale had good reliability.

Empathy was measured using the Toronto Empathy Questionnaire^58^ which primarily focusses on the emotional aspect of empathy. The scale consists of 16 items on a 5-point Likert scale from 0 (*never*) to 4 (*always*). Items 2, 4, 7, 1, 11, 12, 14 and 15 are reverse scored. Scores are totalled with a possible minimum score of 0 and maximum score of 64, with higher scores reflecting higher levels of empathy. An example item includes ‘When someone else is feeling excited, I tend to get excited too’. This scale showed a good level of reliability in analysis run on our data with a Cronbach’s alpha of 0.818.

Personality was assessed using a short form English version^59^ of the Big Five Inventory^60^ (BFI-S) which measures the Big Five personality traits: openness to experience, conscientiousness, extraversion, agreeableness and neuroticism on a short 15-item scale. Each personality trait corresponds to 3 items of the questionnaire along a 7-point Likert scale from 1 (*strongly disagree*) to 7 (*strongly agree*). Items 3, 6, 10 and 14 are reversed. Scores for each trait are summed with higher scores reflecting higher levels of that trait, with each trait having a possible minimum score of 3 and a maximum of 21. An example item corresponding to extraversion includes ‘I see myself as someone who is reserved’. We ran a reliability analysis which produced the following Cronbach’s alpha for each scale: neuroticism = 0.793 (acceptable reliability), extraversion = 0.839 (good reliability), openness = 0.612 (questionable reliability), conscientiousness = 0.652 (questionable reliability) and agreeableness = 0.549 (poor reliability). No modifications were made to the questionnaire as a result of the reliability analysis. Sensitivity analyses were planned excluding traits with questionable or poor reliability (openness to experience, conscientiousness and agreeableness) to examine their effect on the planned analysis.

### Procedure

Participants were invited to complete the survey between November 2019 and December 2019 via social media platforms including Facebook, Twitter and Instagram, and participate by opening the link attached. Participants were offered the opportunity to enter themselves into a prize draw upon completion to win a £50 Amazon voucher. Once clicking the link, participants read background information regarding the study and provided written informed consent. Demographic information was collected including participants’ sex, age and level of education.

Using a design setting on Qualtrics, participants were randomly allocated to a condition in which they would read one of four modified vignettes that were adapted from the Jorm et al.^61^ study. The vignettes described either a hypothetical female or male with depression, or alternatively a hypothetical female or male with chronic back pain. For example, ‘Joe/Ellie is a 21 year-old male/female who has been diagnosed with depression. He/she has been feeling sad and miserable for the last few weeks. He/she can’t focus on his/her studies due to the depression he/she is experiencing, and his/her marks have subsequently dropped. He/she is tired all the time and has trouble sleeping at night. He/she puts off making any decisions and even day‐to‐day tasks seem too much for him/her’. For chronic back pain, the identical vignettes were used but chronic low back pain was used as health condition instead of depression to ensure comparability between conditions. Issues in the vignettes include common symptoms of both conditions with mood and cognitive facets.^62–66^ Functional difficulties such as drop in study marks were selected to illustrate the impact both conditions have on academic work.^64,67^ The vignettes described a young person as we were aware participant recruitment would primarily involve university students and we wanted to ensure participants could identify with the person in the vignette.

Participants were then presented with measures of stigma, empathy and personality. Regarding conditions, 61 participants responded to the female depression vignette, 65 to the female chronic back pain vignette, 62 to the male depression vignette and 65 to the male chronic back pain vignette.

### Design and analysis

A sample size of 98 was calculated using a power analysis G*Power 3 statistical analysis program,^68^ assuming a medium effect size, power of 80%, and aiming at a 0.05 significance level for regression analysis. Nine predictors were entered in total (6 main predictors, 3 control predictors). Therefore, the sample size of 253 satisfied the power calculation requirement.

The present study included two stages of statistical analyses. The first, being an independent groups analysis using a 2x2 independent factorial measures ANOVA, with two independent variables: diagnosis of the person in the vignette (depression or chronic back pain) and sex of the person in the vignette (male or female). The dependent variable was total willingness to interact score. If a significant interaction was found, a post-hoc test would have been carried out by splitting the file by diagnosis, followed by two independent t-tests with a Bonferroni correction of 0.025.

In the second stage, an advanced correlational analysis whereby two hierarchical multiple regressions were run depending on diagnosis of the vignette; the first being for depression and the second being for chronic back pain. Assumptions of the regression model were checked including the assumption of linearity, no multicollinearity, independence, homoscedasticity and multivariate normality. The outcome variable for both regressions was total willingness to interact score, with the predictor variables being empathy, openness to experience, conscientiousness, extraversion, agreeableness and neuroticism, while controlling for sex, age and exposure to mental/physical chronic health conditions. Correlations and t-tests were run for control variables of sex, personal exposure and age with willingness to interact scores prior to entering them into the regression analyses. Control variables were entered together within the first model of the regression.

## Results

The demographic characteristics of participants according to vignette condition are presented in Table 1.

**Table 1.** Participant demographic characteristics according to vignette condition.

Vignette condition		Participant characteristics						
		Mean age (SD.)	Percentage of female participants	Percentage of participants with personal exposure to condition	Education			
					GCSE or equivalent	A level or equivalent	Undergraduate degree or equivalent	Postgraduate degree or equivalent
Depression	Male (*N* = 62)	29.68 (16.30)	56%	66%	6.5%	12.9%	77.4%	3.2%
	Female (*N* = 61)	27.64 (13.45)	70%	54%	3.3%	16.4%	65.6%	14.8%
Chronic pain	Male (*N* = 65)	28.48 (14.28)	72%	25%	7.7%	10.8%	70.8%	10.8%
	Female (*N* = 65)	28.86 (14.61)	69%	25%	7.7%	24.6%	58.5%	9.2%

### Comparisons between depression and chronic back pain vignettes on willingness to interact scores

The assumption of homogeneity of variance was met (*F* (3, 249) = 0.28, *p* = 0.838) as Levene’s test of equality of error variances was not significant. There was no significant difference in willingness to interact scores depending on whether the diagnosis of the person in the vignette had depression or chronic back pain (*F* (1, 249) = 0.68, *p* = 0.410). There was also no significant difference in willingness to interact scores depending on whether the sex of the person in the vignette was male or female (*F* (1, 249) = 0.00, *p* = 0.973). No significant interaction was found between diagnosis and sex of the person in the vignette on willingness to interact scores (*F* (1, 249) = 0.57, *p* = 0.450). See Table 2 for descriptive statistics.

**Table 2.** Descriptive statistics for willingness to interact scores towards vignettes.

Diagnosis of vignette	Sex of vignette	Mean	SD
Depression (*N* = 123)	Male (*N* = 62)	26.73	5.16
	Female (*N* = 61)	26.26	5.44
	Total (*N* = 123)	26.50	5.28
Chronic back pain (*N* = 130)	Male (*N* = 65)	26.77	4.95
	Female (*N* = 65)	27.28	4.86
	Total (*N* = 130)	27.02	4.89

### Participants’ empathy and personality as predictors of willingness to interact towards depression

For social exclusion towards depression, the regression assumptions were met. The zero order correlations for all variables were within acceptable limit of −0.9 and 0.9. Tolerance values were all greater than 0.20 and all VIF statistics less than 10. The residuals were normally distributed, and the accuracy of prediction appears similar across scores. There were 3 outliers, equating to 2.44%, meaning the model was robust. See Table 3 for Pearson’s correlations and Table 4 for descriptive statistics.

**Table 3.** Pearson’s correlation analyses for total willingness to interact scores towards depression and chronic back pain, age, empathy, openness, conscientiousness, extraversion, agreeableness and neuroticism.

Diagnosis of vignette		Pearson’s correlation analyses							
		(1)	(2)	(3)	(4)	(5)	(6)	(7)	(8)
Depression (*N* = 123)	Willingness to interact score (1)	—	0.06	0.35***	0.13	−0.14	−0.02	0.23**	0.20*
	Age (2)	—		0.10	0.03	0.32***	0.11	0.23**	0.21**
	Empathy (3)	—			0.33***	0.19*	0.22**	0.54***	0.15
	Openness (4)	—				0.07	0.07	0.16*	0.01
	Conscientiousness (5)	—					0.14	0.26**	0.16*
	Extraversion (6)	—						0.08	0.35***
	Agreeableness (7)	—							0.01
	Neuroticism (8)	—							
Chronic back pain (*N* = 130)	Willingness to interact score (1)	—	0.04	0.42***	0.19*	0.13	0.06	0.15*	0.11
	Age (2)	—		0.08	0.17*	0.29***	0.11	0.06	0.14
	Empathy (3)	—			0.26***	0.26***	0.19*	0.49***	0.16*
	Openness (4)	—				0.08	0.27***	0.08	0.05
	Conscientiousness (5)	—					0.26**	0.33***	0.16*
	Extraversion (6)	—						0.11	0.29***
	Agreeableness (7)	—							0.05
	Neuroticism (8)	—							

*p ≤ 0.05, **p ≤ 0.01, ***p ≤ 0.001.

**Table 4.** Descriptive statistics for willingness to interact scores, male and female participants willingness to interact scores, age, empathy, openness, conscientiousness, extraversion, agreeableness and neuroticism.

Variable	Descriptive statistics			
	Depression vignette (*N* = 123)		Chronic pain vignette (*N* = 130)	
	Mean	SD.	Mean	SD.
Willingness to interact	26.50	5.28	27.02	4.89
Male participants	26.42	5.07	25.80	4.45
Female participants	26.54	5.43	27.53	4.99
Age	28.67	14.93	28.67	14.36
Empathy	48.17	6.79	47.52	6.61
Openness	15.59	2.69	15.50	3.05
Conscientiousness	15.46	2.93	15.21	3.43
Extraversion	13.65	4.03	13.52	4.12
Agreeableness	15.31	3.13	14.85	3.04
Neuroticism	12.74	4.24	13.55	4.11

Willingness to interact scores did not significantly differ according to participant sex (*p* = 0.454) or personal exposure (*p* = 0.097) and did not correlate with participant age (*p* = 0.244). When used as control variables, personal exposure, sex and age explained 2.1% of the variance in willingness to interact, these predictors were not significantly associated with willingness to interact (*F* (3, 119) = 0.83, *p* = 0.479). The predictors (empathy and personality) explained a further 20.6% of the variance. Adding the predictors, significantly improved the prediction of the model, over and above the confounds (*F* (6, 113) = 5.01, *p* < 0 .001). The final model was significant (*F* (9, 113) = 3.68, *p* < 0 .001) explaining 16.5% of the variance in willingness to interact. Regarding individual predictors (see Table 5), empathy was significantly positively associated with willingness to interact, and conscientiousness was significantly negatively associated with willingness to interact. In addition, sex was significantly associated with willingness to interact indicating that females showed less willingness to interact than males. However, inconsistencies were distinguished between the coefficient table (see Table 5) compared to the descriptive statistics (see Table 4), which suggested that females were significantly associated with greater willingness to interact than males. A t test was also run to examine this further, but it was non-significant. Age of participant, personal exposure, neuroticism, extraversion, openness and agreeableness were not significantly associated with willingness to interact towards the depression vignette. Pearson’s correlation analysis (Table 3) suggested agreeableness was significantly associated with both willingness to interact (0.23) and empathy (0.54), but it was not a significant predictor in the regression analysis.

**Table 5.** Hierarchical regression analyses for willingness to interact scores.

Diagnosis of vignette	Variable	Simultaneous				
		*B* (SE.)	Beta	*t*	*p*	95% CIs
Depression (*N* = 123)	Step 1					
	Personal exposure	1.391 (0.99)	0.13	1.4	0.162	−0.57, 3.35
	Sex	0.067 (1.00)	0.01	0.1	0.946	−1.90, 2.04
	Age	−0.029 (0.03)	−0.08	−0.9	0.370	−0.09, 0.04
	Step 2					
	Personal exposure	0.936 (0.96)	0.09	1.0	0.333	−0.97, 2.84
	Sex	−2.260 (1.0)	−0.21	−2.2	0.031	−4.31, −0.21
	Age	−0.014 (0.03)	−0.04	−0.4	0.667	−0.08, 0.05
	Empathy	0.300 (0.09)	0.39	3.4	0.001	0.12, 0.48
	Openness to experience	−0.019 (0.18)	−0.01	−0.1	0.913	−0.37, 0.33
	Extraversion	−0.034 (0.12)	−0.03	−0.3	0.783	−0.28, 0.21
	Conscientiousness	−0.350 (0.16)	−0.19	−2.1	0.034	−0.67, −0.03
	Agreeableness	0.219 (0.17)	0.13	1.3	0.209	−0.12, 0.56
	Neuroticism	0.166 (0.13)	0.13	1.3	0.193	−0.09, 0.42
Chronic back pain (*N* = 130)	Step 1					
	Personal exposure	1.790 (1.02)	0.16	1.8	0.080	−0.22, 3.80
	Sex	1.634 (0.93)	0.15	1.8	0.082	−0.21, 3.5
	Age	−0.002 (0.03)	−0.01	−0.1	0.942	−0.06, 0.06
	Step 2					
	Personal exposure	1.445 (0.98)	0.13	1.5	0.143	−0.50, 3.39
	Sex	1.107 (0.94)	0.10	1.2	0.244	−0.76, 2.98
	Age	−0.015 (0.03)	−0.04	−0.5	0.636	−0.08, 0.05
	Empathy	0.332 (0.08)	0.45	4.5	0.000	0.19, 0.48
	Openness to experience	0.172 (0.15)	0.11	1.2	0.250	−0.12, 0.47
	Extraversion	−0.213 (0.11)	−0.18	−2.0	0.051	−0.43, 0.00
	Conscientiousness	0.056 (0.14)	0.04	0.4	0.684	−0.22, 0.33
	Agreeableness	−0.136 (0.15)	−0.09	−0.9	0.376	−0.44, 0.17
	Neuroticism	−0.049 (0.11)	−0.04	−0.5	0.646	−0.26, 0.16

### Participants’ empathy and personality as predictors of willingness to interact towards chronic back pain

For social exclusion towards chronic back pain, the regression assumptions were met. The zero order correlations for all variables were within acceptable limit of −0.9 and 0.9. Tolerance values were all greater than 0.20 and all VIF statistics less than 10. The residuals were normally distributed and the accuracy of the predications appear to be similar across scores. There were 4 outliers, equating to 3.08% of the sample meaning the model was robust. See Table 3 for Pearson’s correlations and Table 4 for descriptive statistics.

Female participants (*p* = 0.032) and participants with personal exposure to chronic pain (*p* = 0.028) were significantly more willing to interact with the hypothetical individual. Age did not significantly correlate with willingness to interact scores (*p* = 0.327). When used as control variables, personal exposure, sex and age explained 5.1% of the variance in willingness to interact, these variables were not significantly associated with willingness to interact (*F* (3, 126) = 2.28, *p* = 0.083). The predictors (empathy and personality) explained a further 19.3% of the variance. Adding the predictors, significantly improved the prediction of the model, over and above the confounds (*F* (6, 120) = 5.11, *p* < 0 .001). The final model was significant (*F* (9, 120) = 4.31, *p* < 0 .001) explaining 18.8% of the variance in willingness to interact. Regarding individual predictors (see Table 5), empathy was significantly positively associated with willingness to interact. Age and sex of participants, personal exposure, extraversion, neuroticism, conscientiousness, openness and agreeableness were not significantly associated with willingness to interact towards the chronic back pain vignette. Pearson’s correlation analysis (Table 3) suggested willingness to interact was also significantly associated with openness (0.19) and agreeableness (0.15), which were not significant predictors in the regression analysis. Empathy (significant predictor) was significantly correlated with both openness (0.26) and agreeableness (0.49).

### Sensitivity analyses

Sensitivity analyses were run excluding traits with questionable or poor reliability (openness to experience, conscientiousness and agreeableness) to examine their effect on the planned analysis.

For the depression vignette, the control variables of personal exposure, sex and age explained 2.1% of the variance in willingness to interact, these predictors were not significantly associated with willingness to interact (*F* (3, 119) = 0.83, *p* = 0.479). The predictors (empathy, extraversion and neuroticism) explained a further 16.8% of the variance in willingness to interact. Adding the predictors, significantly improved the prediction of the model, over and above the confounds (*F* (3, 116) = 7.98, *p* <0 .001). The final model was significant (*F* (6, 116) = 4.48, *p* < 0 .001) explaining 14.6% of the variance in willingness to interact. Regarding individual predictors, sex (*t* = −2.1, Beta = −0.20, *p* = 0.039) and empathy (*t* = 4.4, Beta = 0.42, *p* < 0.001) were significantly associated with willingness to interact.

For the chronic back pain vignette, the control variables of personal exposure, sex and age explained 5.1% of the variance in willingness to interact, these predictors were not significantly associated with willingness to interact (*F* (3, 126) = 2.28, *p* = 0.083). The predictors (empathy, extraversion and neuroticism) explained a further 17.8% of the variance in willingness to interact. Adding the predictors, significantly improved the prediction of the model, over and above the confounds (*F* (3, 123) = 9.45, *p* < 0 .001). The final model was significant (*F* (6, 123) = 6.09, *p* < 0 .001) explaining 19.2% of the variance in willingness to interact. Regarding individual predictors, empathy (*t* = 5.2, Beta = 0.44, *p* < 0.001) was significantly associated with willingness to interact.

## Discussion

The present study aimed to investigate whether social exclusion, through measurement of willingness to interact, differed towards hypothetical males and females with depression or chronic back pain, and whether the participant’s empathy and personality traits were associated with social exclusion. No significant differences were found in willingness to interact scores depending on the diagnosis of the hypothetical person in the vignette. This suggests that people with depression and chronic back pain face similar levels of social exclusion regardless of their condition, refuting the hypotheses and previous research which anticipated greater stigma towards depression.^30^ This could be due to chronic back pain often being perceived as a common and trivial medically unexplained condition,^21,27,69^ attracting high levels of public stigma.^9^ People with chronic back pain often report that their pain and its impact on their lives is disbelieved by others.^33^ It is possible that depression and chronic back pain elicited similar social exclusion from respondents as both are commonly comorbid conditions which cause disabling symptoms.^70–72^ Scott et al.^31^ recently reported that experiences of discrimination in those with pain are associated with increased depression and loneliness.

One may also consider whether a lack of significant difference in willingness to interact scores between diagnoses could be accounted for by alternative factors. For example, our vignettes described young adults who are often perceived as agile, physically healthy and rarely affected by chronic pain, which may have contributed to the non-significant difference between depression and chronic pain stigma. Participants may have also responded to different levels/types of depression as a general term of ‘depression’ was used in the vignette. This is likely considering that diagnostic labelling affects lay people’s attitudes towards mental illness, for example, depression is perceived as less severe than schizophrenia.^73^ Alternatively, if a more generalised concept of ‘mental illness’ would have been described in the vignette, it is likely stronger stigmatising views from participants would have been elicited as research reports that labelling a vignette as a ‘mental illness’ increases participant’s desire to socially distance from such individual compared to when described as having depression alone.^74^

No significant differences were found in willingness to interact scores depending on the sex of the vignette, implying that males and females with depression and chronic back pain face similar levels of social exclusion. This contradicts the hypothesis and previous mental health research which found greater stigma exists towards males.^32^ However, within this past research, participants provided explanations towards their stigma. For example, participants blamed males more for their condition and believed symptoms were their own fault indicating elements of blame rather than social exclusion. The present study did not allow participants to expand on their thoughts, potentially eluding differences in these findings.

Empathy was largely implicated in the social exclusion process, with empathy being significantly associated with greater willingness to interact towards both the depression and chronic back pain vignettes. Thus, suggesting empathy is a focal trait in stigmatising attitudes. This supports the hypothesis and previous research^35–37^ extending this relationship into a social exclusion dimension of stigma in the general population. Findings highlight the importance of increasing empathy to reduce stigma in society. Supplementary research has shown that it is possible to increase levels of empathy. Stepien and Baernstein^75^ peer-reviewed 13 studies and concluded that is it possible to use interventional strategies to create a positive change in empathy. For example, communication skill workshops were observed to be the most effective intervention in increasing participant’s behavioural empathy. As individuals with chronic back pain often report feeling guilty, judged, blamed and intimidated by health professionals, friends, family and colleagues,^19–22^ findings raise the posssibility of using communication skill workshops in these groups to reduce social exclusion towards people with chronic back pain. Consequently, improving well-being and recovery for this population.

Higher conscientiousness associated with less willingness to interact towards the hypothetical individual with depression, supporting past research which found that the more conscientious participants were, the more they desired increased social distance from those with mental health conditions.^41^ However, previous studies have not found this effect with other dimensions of stigma suggesting conscientiousness is specifically associated with a social exclusion dimension of stigma towards mental health.^39,41^ Conscientious individuals tend to behave in self-controlled ways which follow socially prescribed norms with orderly and rule abiding manners.^76^ It is possible that mental illnesses, including depression, are seen as out of societal norms for those with high conscientiousness meaning they desire greater social distance from such individuals to follow the socially prescribed norms.

For chronic back pain, none of the personality traits were significant predictors of social exclusion, however, extraversion approached significance (*B***=** −0.213, *p* < 0.051) with greater levels of extraversion being associated with less willingness to interact. This resembles previous findings from mental health literature which found that extraversion is associated with ‘weak-not-sick’ beliefs, suggesting extraversion is influential in stigma.^41^ Extraversion has been shown to increase one’s capacity to effectively manage chronic pain with increased pain thresholds and greater pain tolerance in individuals with higher levels of extraversion.^77,78^ Therefore, it is possible that extraverted individuals express greater social exclusion towards those with chronic back pain as they respond differently to pain themselves, creating a barrier to understanding another’s chronic pain.

Personality is a fairly novel area of research in this specific area, with former studies emphasising the influence of openness to experience and agreeableness on stigma towards mental health.^39–41^ In the present study, neither trait was associated with willingness to interact scores in the regression model towards hypothetical individuals with depression or chronic back pain, contradicting previous research and the hypothesis. However, willingness to interact scores significantly correlated with agreeableness in both vignette conditions, and openness to experience with willingness to interact scores in the chronic back pain condition. Both, openness to experience and agreeableness were associated with empathy too (significant predictor in both vignette conditions), suggesting an interaction between these predictors in the regression analyses.

Unexpected findings for openness and agreeableness may also to an extent be a result of the measure of personality used, with analysis suggesting an unacceptable level of reliability in our study, despite research suggesting that the English version of the scale had acceptable validity and reliability.^59^ However, results were similar when running a sensitivity analysis without the personality traits with poor reliability. Brief measures, like the BFI-S used within this study, are often criticised for having reliability issues^79^ with other shortened versions of measures assessing the Big Five Personality traits having concerning differences in internal reliability across personality traits.^80^ Interestingly, in an assessment of the BFI-S German version, agreeableness fell short on internal consistency compared to other traits,^81^ as seen within this study. This suggests the concept of agreeableness was not likely measured effectively and hence contradicted previous findings.

It should also be considered that this study examined a discriminatory behaviour aspect of public stigma termed social exclusion. Considering that public stigma is a broad construct, perhaps examination of other dimensions of stigma such as beliefs or emotional reactions may elicit stronger associations with the personality traits, particularly regarding agreeableness and openness to experience which have shown associations with stigma in previous literature.^39–41^ Alternatively, it may be that social exclusion as a behaviour towards illness is not related to traits of openness or agreeableness in the general population.

Despite not all control variables having significant relationships with willingness to interact scores, these variables were still included within the regression analyses as planned due to past research supporting the relationship between these variables and stigma. The confounding variables were non-significant; except for sex, where the regression results showed that females had greater social exclusion scores towards the hypothetical individual with depression than males. However, a follow up analysis showed that this difference was non-significant; indicating that the regression result is likely to be an outcome of the interaction between predictors within the model. Participants’ sex was not significantly associated with stigma towards the chronic back pain vignette, supporting previous research which found no significant differences in stigma of female and male nursing students towards chronic pain conditions.^53^ The present study therefore extends this finding to the general population beyond a medical sample. However, the present study’s sample had a greater number of female and young participants which might have influenced the results for sex and age. Additionally, the question about personal exposure to mental/physical chronic health conditions was rather generic and did not specifically ask about depression or chronic back pain. It is possible participants responded to varying levels of personal exposure, limiting our understanding of this control variable.

The present study examined a poorly explored research question of comparing social exclusion towards mental and physical health conditions in the general population. Further development of effective interventions to reduce mental health and chronic pain stigma are essential to aid recovery for both patient groups.^9,82^ However, a key limitation of the present study is the use of vignette methodology. Vignettes allow researchers to create elaborate stimulus to be used in random assessment and hypothesis testing. Yet concerns have been raised regarding whether this measure accurately reflects real life as measurements mirror how respondents would behave in a hypothetical scenario.^83^ However, additional research suggests there is significant concordance between hypothetical scenarios and actual behaviours.^84^

This research also raises concerns with social desirability bias as stigma is considered to be a negative response towards an individual, meaning the likelihood participants’ responses will reflect their true feelings is questionable .^3^ Nevertheless, one study demonstrated that asking stigma related questionnaires via a computer (the method used in our study) in comparison to an interview reduces the effects of social desirability.^85^

This research did not collect data on participant’s language fluency, race or ethnicity. A recent systematic review reported differences in public stigma between ethnic minority and majority groups,^86^ with our lack of ethnic data limiting our understanding of how the ethnic background of our sample may have affected stigma scores.

In addition, it was assumed that the stigma scale,^32^ originally created for mental health stigma, was also appropriate for measuring stigma towards chronic pain due to the nature of the questions. This assumption allowed stigma scores to be directly compared between conditions, but it must be considered that non-significant results may have arisen from the operationalisation of stigma or an inability to measure chronic pain stigma effectively. Van Brakel^87^ also highlighted this issue, suggesting a reliable and valid generic scale for different health conditions, including mental health and chronic pain conditions, does not exist with most scales being condition specific. This indicates an on-going need for researchers to develop a stigma scale appropriate for both mental health and chronic pain conditions. Additionally, a continuous total score of participant’s willingness to interact was used in analyses, and whilst using Likert total scores is a widely accepted method to measure concepts in a reliable manner,^88^ indeed there is controversy whether it is appropriate to convert ordinal data into numbers and analyse this as interval data. Using a continuous total means the results may not represent participant’s overall willingness to interact in the most appropriate form. We did not dichotomise participant’s scores into willing and unwilling to interact due to the continuous nature of the scale, with dichotomizing potentially resulting in misrepresentation of participant’s original answer.

The present study contributes to previous stigma research suggesting that males and females with depression or chronic back pain face similar levels of stigma. Therefore, interventions to reduce stigma should be equally focused towards both groups. Findings also established that empathy plays a key role in stigmatising attitudes, suggesting empathy potentially acts as an underlying mechanism influencing stigma. Conscientiousness was the only personality trait associated with willingness to interact towards depression. The knowledge from this research improves understanding of the variables which exacerbate stigmatising attitudes. Therefore, findings can assist with the well-needed development of societal intervention programmes to reduce stigma and promote recovery for those facing difficult and challenging health conditions.
